# Two novel monoclonal antibodies against fiber-1 protein of FAdV-4 and their application in detection of FAdV-4/10

**DOI:** 10.1186/s12917-019-1987-5

**Published:** 2019-07-08

**Authors:** Hongxia Shao, Yanan Lu, Weikang Wang, Tuofan Li, Jianjun Zhang, Zhimin Wan, Guangchen Liang, Wei Gao, Aijian Qin, Jianqiang Ye

**Affiliations:** 1 Key Laboratory of Jiangsu Preventive Veterinary Medicine, Key Laboratory for Avian Preventive Medicine, Ministry of Education, College of Veterinary Medicine, Yangzhou University, Yangzhou, 225009 Jiangsu China; 2 Jiangsu Co-innovation Center for Prevention and Control of Important Animal Infectious Diseases and Zoonoses, Yangzhou, 225009 Jiangsu China; 3 Joint International Research Laboratory of Agriculture and Agri-Product Safety, the Ministry of Education of China, Yangzhou University, Yangzhou, 225009 Jiangsu China; 4 Sinopharm Yangzhou VAC Biological Engineering Co. Ltd, Yangzhou, 225127 Jiangsu China; 5 Institutes of Agricultural Science and Technology Development, Yangzhou University, Yangzhou, 225009 Jiangsu China

**Keywords:** FAdV-4, Fiber-1, mAb, IFA, Immunoprecipitation, Sandwich ELISA

## Abstract

**Background:**

Recently, serotype 4 fowl adenovirus (FAdV-4) has spread widely and caused huge economic loss to poultry industry. However, little is known about the molecular pathogenesis of FAdV-4. Fiber protein is thought to be vital for its infection and pathogenesis.

**Results:**

Two novel monoclonal antibodies (mAbs) targeting the fiber-1 protein of FAdV-4 were generated, designated as mAb 3B5 and 6H9 respectively. Indirect immunofluorescence assay (IFA) showed that both mAbs only reacted with the FAdV-4 and FAdV-10, not with other serotypes including FAdV-1, FAdV-5, FAdV-6, FAdV-7, FAdV-8 and FAdV-9 tested. Although both mAbs did not recognize the linear epitopes, they could efficiently immunoprecipitate the fiber-1 protein in LMH cells either infected with FAdV-4 or transfected with pcDNA3.1-Fiber-1. Moreover, mAb 3B5 as a capture antibody and HRP-conjugated mAb 6H9 as a detection antibody, a novel sandwich ELISA for efficient detection of FAdV-4 was generated. The limit of detection of the ELISA could reach to 1000 TCID_50_/ml of FAdV-4 and the ELISA could be efficiently applied to detect FAdV-4 in the clinical samples.

**Conclusion:**

The two mAbs specific targeting fiber-1 generated here would pave the way for further studying on the role of fiber-1 in the infection and pathogenesis of FAdV-4, and the established mAb based sandwich ELISA would provide an efficient diagnostics tool for detection of FAdV-4/10.

## Background

Fowl adenovirus (FAdV) belongs to the family *Adenoviridae*, genus *Aviadenovirus* [1]. Based on its genome sequence and sera cross-neutralization, FAdV is currently clustered into 5 species (FAdV-A to FAdV-E) with 12 serotypes (FAdV-1 to -8a and FAdV-8b to − 11) [1, 2]. The diseases caused by the infection of FAdV mainly include inclusion body hepatitis (IBH), hepatitis-hydropericardium syndrome (HPS), and gizzard erosion and ulceration (GEU) [2–4]. Disease of IBH and HPS in chicken flocks has been spread globally, particularly in Eurasian. Among these serotypes, serotype FAdV-2, 8a, 8b and 11 can induce IBH, whereas serotype FAdV-4 is the main causative agent for HPS [5]. Recently, the spread of HPS caused by FAdV-4 has resulted in huge economic loss to poultry industry [5]. Notably, the emerging of the highly pathogenic FAdV-4 with novel genotype and broad host range in China called for more efficient control strategies for the FAdV-4 [6–12]. The binding of the viral protein with host receptor is critical for initiating the viral infection. Hexon, penton and fiber are three major proteins on the surface of viral particle of the adenovirus [1]. During the infection, the fiber protein, but not the hexon and penton, can directly bind to the viral receptor [1]. It should be noted that serotypes FAdV-1, FAdV-4 and FAdV-10 carry two fiber proteins, designated as fiber-1 and fiber-2 [1]. Previous studies demonstrate that both fiber proteins of FAdV-4 play significant roles in the viral infection and pathogenesis and they carry dominant B cell epitopes which can differentiate FAdV-4 from other serotypes of FAdV [13–15]. Since the commercial monoclonal antibody (mAb) against the fiber proteins of FAdV-4 is not available, the molecular mechanism for its infection and pathogenesis of FAdV-4 is largely unknown, and the efficient diagnostics for FAdV-4 is also available. In this study, two novel mAbs specific to the fiber-1 protein of FAdV-4 were generated. Both mAbs could efficiently immunoprecipitate the fiber-1 protein either in the infected cells or the transfected cells. Moreover, a mAb based sandwich ELISA for specific detection of FAdV-4/10 was established.

## Results

### Two mAbs specific to fiber-1 of FAdV-4 were generated

**Fig. 1:**
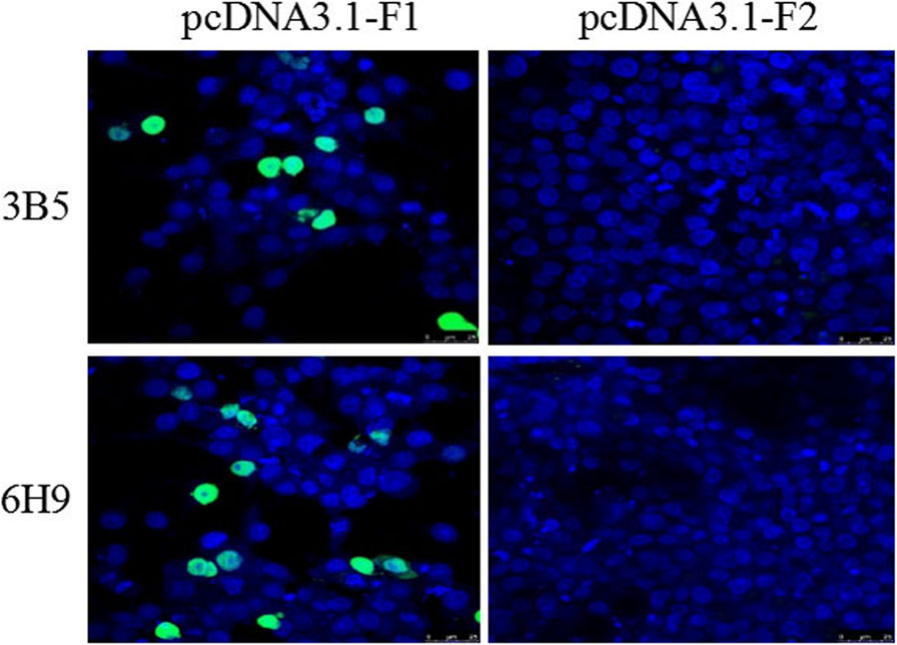
Recognizing fiber-1 of FAdV-4 by the two mAbs in IFA mAb 3B5 and 6H9 reacted with the LMH cells transfected with pcDNA3.1-F1 and pcDNA3.1-F2 expressing fiber-1 and fiber-2 of FAdV-4 respectively. The FITC-conjugated goat anti-mouse IgG was used as secondary antibody

**Fig. 2:**
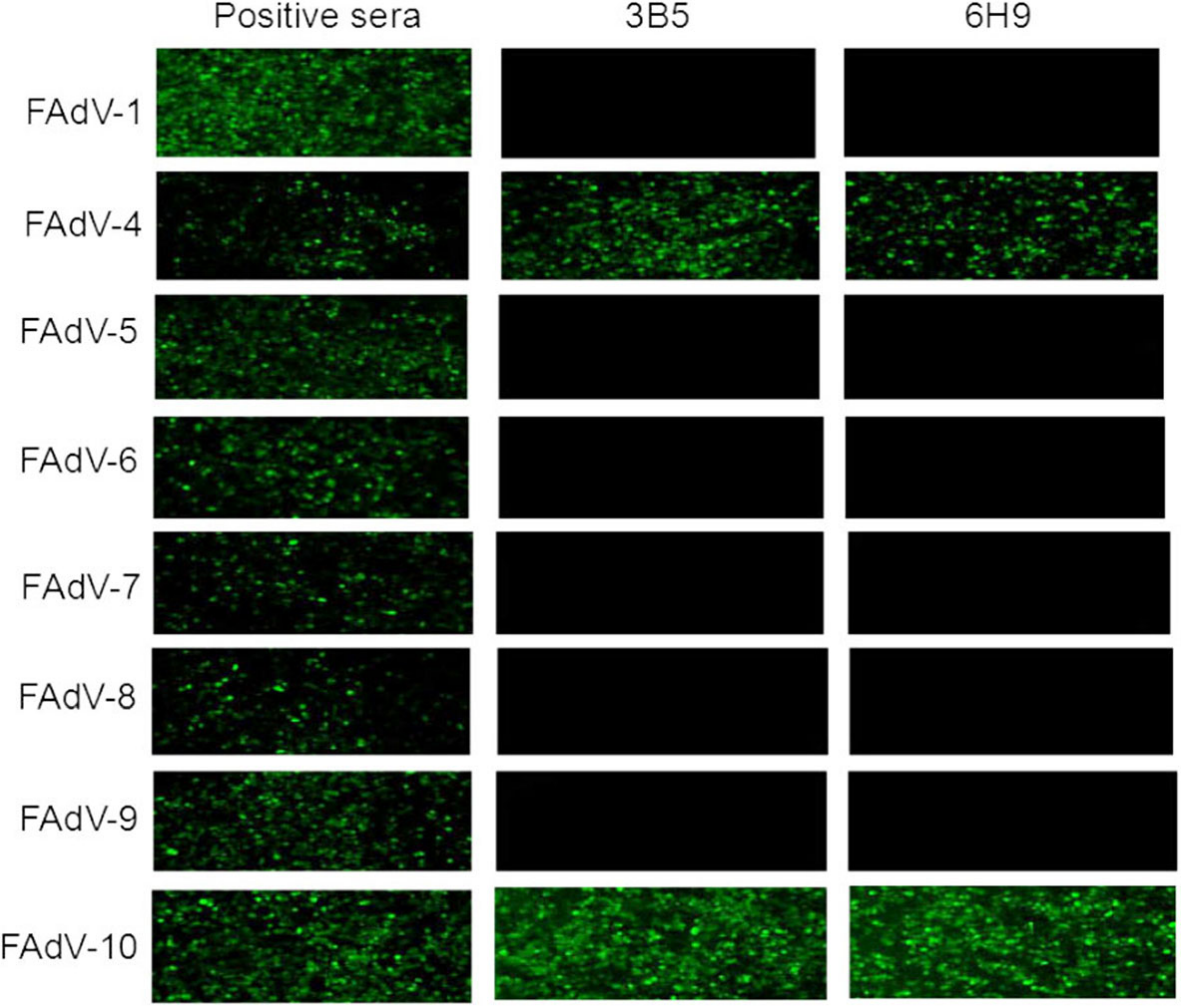
Cross-reaction for mAb 3B5 and 6H9 against different FAdV viruses in IFA mAb 3B5 and 6H9 reacted with the LMH cells infected with different serotype FAdV viruses including FAdV-1, FAdV-4, FAdV-5, FAdV-6, FAdV-7, FAdV-8, FAdV-9 and FAdV-10 respectively. The FITC-conjugated goat anti-mouse IgG was used as secondary antibody

Balb/c mice were immunized with purified prokaryotic fusion protein GST-F1-S and LMH cells infected with FAdV-4 were used as a screening antigen for mAbs. Through IFA screening, two hybridomas cell strains stably secreting mAb against FAdV-4 were generated, named as 3B5 and 6H9 respectively. Moreover, both mAbs could efficiently react with the LMH cells transfected with pcDNA3.1-F1, but not with pcDNA3.1-F2 as described in Fig. 1. To investigate the specificity for the two mAbs, different serotypes of fowl adenoviruses were used to be tested in IFA. As shown in Fig. 2, mAb 3B5 and 6H9 could not react with the LMH cells infected with FAdV-1, FAdV-5, FAdV-7, FAdV-8 and FAdV-9 tested while they could efficiently react with the LMH cells infected with FAdV-4 and FAdV-10. Notably, both FAdV-4 and FAdV-10 belong to FAdV-C species carrying similar fiber-1 protein. Therefore, the two mAbs showed great specificity to FAdV-C species, indicating the application for differentiating FAdV-C from other species of FAdV.

### mAb 3B5 and 6H9 could efficiently immunoprecipitate fiber-1 of FAdV-4

**Fig. 3:**
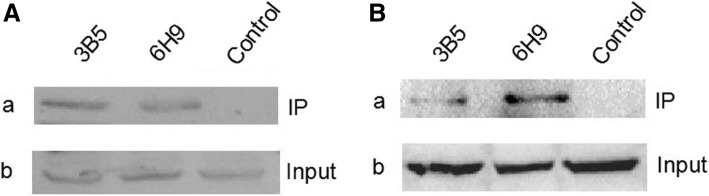
Immunoprecipitation assay for mAb 3B5 and 6H9 **a** mAb 3B5 and 6H9, but not the control mAb, could immunoprecipitate the fiber-1 protein (about 48kD) in LMH cells infected with FAdV-4. a and b, Western blot assay for the immunoprecipitated pellets or the lysates of the infected cells using chicken sera against FAdV-4 respectively; **b** mAb 3B5 and 6H9, but not the control mAb, could immunoprecipitate the fiber-1 protein (about 48kD) in LMH cells transfected with pcDNA3.1-F1. a and b, Western blot assay for the immunoprecipitated pellets or the lysates of the transfected cells using chicken sera against FAdV-4 respectively

To further characterize the two mAbs generated here, LMH cells infected with FAdV-4 or transfected with pcDNA3.1-F1 were analyzed through Western blot and immunoprecipitation. Although both mAbs did not recognize the linear epitopes in Western blot assay, they could efficiently immunoprecipitate the fiber-1 protein in LMH cells either infected with FAdV-4 or transfected with pcDNA3.1-F1. As described in Fig. 3, after the immunoprecipitation of the lysates from LMH cells either infected with FAdV-4 or transfected with pcDNA3.1-F1 using mAb 3B5 and 6H9, a specific protein band with the size of 48kD could be found in the immunoprecipitated pellets in the Western blot by using a chicken polyclonal antibody against FAdV-4, whereas such protein band could not be found when using the control mAb. This data demonstrated that mAb 3B5 and 6H9 could efficiently recognize the natural or conformational epitopes in the fiber-1 protein of FAdV-4.

### A novel mAbs based sandwich ELISA for detection of FAdV-4/10 was developed

**Fig. 4:**
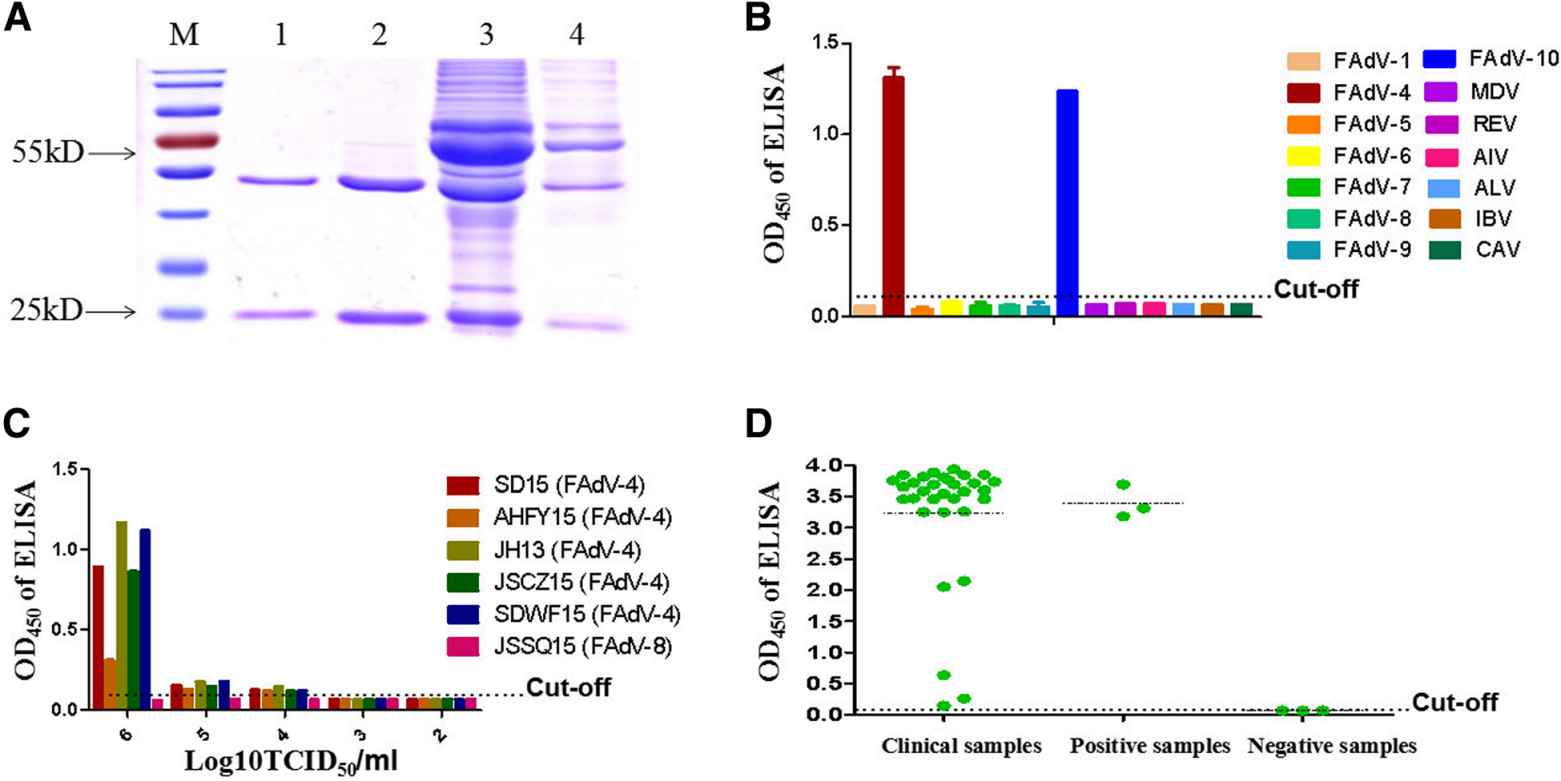
A novel mAbs based sandwich ELISA for efficient detection of FAdV-4/10 **a** SDS-PAGE assay for the purified mAb 3B5 and 6H9. Lane 1 and lane 2: the purified mAb 3B5 and 6H9 respectively; Lane3 and lane 4: the unpurified mAb 3B5 and 6H9 respectively; **b** Specificity assay for the sandwich ELISA using different viruses including FAdV-1, FAdV-4, FAdV-5, FAdV-6, FAdV-7, FAdV-8, FAdV-9, FAdV-10, MDV, AIV, ALV, REV, IBV and CAV; **c** Sensitivity assay for the sandwich ELISA using different doses of different FAdV-4 isolates; **d** Detection of FAdV-4 in 32 clinical liver samples using the sandwich ELISA. Three liver samples from the SPF chickens were used as negative control and three liver samples from the chickens experimentally infected with FAdV-4 were used as positive control

Since both mAbs could efficiently capture the natural fiber-1 protein of FAdV-4, to evaluate whether the two mAbs 3B5 and 6H9 could be applied to generate a sandwich ELISA for specific detection of FAdV-4, the purifed mAbs were labeled with HRP. As shown in Fig. 4-a, the heavy chain and light chain of the purified mAbs 3B5 and 6H9 could be found in the SDS-PAGE analysis. After conjugated with HRP and optimal selection, the mAb 6H9-HRP was used as detection antibody and the purified mAb 3B5 was selected as capture antibody. Since the arithmetric mean OD_450_ value of the six FAdV-4 free samples was 0.0584, and the cut-off was defined as the two-fold of the mean of the negative samples. Therefore, the OD_450_ value for the cut-off of the ELISA was determined as 2 × 0.0584 = 0.1168. Specificity analysis demonstrated that the ELISA reacted only with the FAdV-4 and FAdV-10 with high OD_450_ value, but not with serotypes FAdV-1, FAdV-5, FAdV-6, FAdV-7, FAdV-8 and FAdV-9 tested as shown in Fig. 4-b. Notably, the ELISA did not show cross-reaction with other viral pathogens such as MDV, AIV, ALV, REV, IBV and CAV tested. Sensitivity analysis further showed that the limit of detection (LOD) of the ELISA was 10^4^ TCID_50_/ml of FAdV-4. As shown in Fig. 4-c, the OD_450_ of the different FAdV-4 strains with dose of 10^4^ TCID_50_/ml or higher doses were all higher than the cut-off value whereas those of the FAdV-8 tested were all less than the cut-off value. Moreover, the ELISA could be efficiently used to detect the FAdV-4 in the clinical samples. As shown in Fig. 4d, all the thirty two liver samples from the diseased chicken flock naturally infected with FAdV-4 were positive in the ELISA. Except for three samples with low OD_450_ value, all other samples showed high OD_450_ value. This ELISA data for these clinical samples was further confirmed by PCR for amplifying the specific band of FAdV-4 (data not shown).

## Discussion

The hepatitis-hydropericardium syndrome induced by FAdV-4 infection has caused huge economic losses to the poultry industry globally. However, the molecular mechanism for the infection and pathogenesis of FAdV-4 need to be elucidated. Although ELISA and PCR for detection of antibody and DNA respectively have been reported for FAdV-4 [6, 17, 18], the rapid and efficient ELISA for detection of FAdV-4 virus was not available so far. As the surface protein of the viral particle, fiber protein plays vital roles in the viral infection and contains dominant B cell epitopes which could differentiate different species or serotypes of FAdV. In our previous report, a novel monoclonal antibody (3C2) against fiber-2 of FAdV-4 could not only immunoprecipitate fiber-2 protein, but also could efficiently inhibit the viral replication of FAdV-4 in vitro [16]. That study indicated that fiber-2 of FAdV-4 carries B cell epitopes for neutralizing the infection of FAdV-4. Here, two mAbs against the fiber-1 of FAdV-4, designated as 3B5 and 6H9, were generated using a purified GST fusion protein with the shaft domain of the fiber-1 of FAdV-4 as immunogen. Although both mAbs could not recognize the linear epitopes of fiber-1 and also did not show neutralizing activity against FAdV-4, they could efficiently immunoprecipitate the fiber-1 from the LMH cells either infected with the FAdV-4 or transfected with pcDNA3.1-F1 plasmid, highlighting their application in identifying the cellular proteins for interacting with the fiber-1 of FAdV-4. Notably, a sandwich ELISA based on the two mAbs were developed for specific detection of FAdV-4/10. Except for the high specificity to FAdV-4/10, the limit of detection of the ELISA could reach to 1000 TCID_50_/ml of FAdV-4 and could be efficiently applied in detecting FAdV-4 in the clinical tissue samples, highlighting the feasibility of the ELISA. Since FAdV-10 and FAdV-4 belong to the same species and the fiber proteins from the two serotypes are highly homologous, the cross-reaction with FAdV-10 for the ELISA was also expected which was consistent with the IFA result. This cross-reaction with FAdV-10 is also consistent with the previous finding reported by Feichtner et al when a fiber-2 based ELISA was used to detect the antibody against FAdV-4 [17]. All these studies suggested that FAdV-4 and FAdV-10 could be clustered into one serotype based on the fibers. Moreover, unlike FAdV-4, the infection of FAdV-10 was rarely happened in the field and FAdV-10 was known as a kind of low pathogenic FAdV.

## Conclusions

To our knowledge, this is the first demonstration of the generation of mAbs against fiber-1 of FAdV-4 and a novel mAbs based sandwich ELISA for efficient detection of FAdV-4/10. The two mAbs specific targeting fiber-1 of FAdV-4 developed here would pave the way for further studying on the roles of fiber-1 in the infection and the identification of the cellular receptor, and provide novel and efficient diagnostics tools for detection of FAdV-4/10.

## Methods

### Viruses and clinical samples

Serotype 1, 5, 6, 7, 9 and 10 fowl adenoviruses (FAdV-1, FAdV-5, FAdV-6, FAdV-7, FAdV-9 and FAdV-10) were from ATCC. Serotype 8 fowl adenovirus (FAdV-8), Marek’s disease virus (MDV), avian reticuloendotheliosis virus (REV), Infectious bronchitis virus (IBV), chicken infectious anemia virus (CAV), H9N2 avian influenza virus (AIV) and subgroup J avian leukosis virus (ALV) were kept in our laboratory. The Serotype 4 fowl adenovirus FAdV-4 strains SD15, SDWF15, AHFY15, JSCZ15 and JH13 were isolated as previously described [6]. Thirty two liver samples of chickens were from a diseased chicken flock naturally infected with FAdV-4. All the studies associated with the viruses were performed in cabinet under ABSL2 facility.

### Cells and plasmids

The LMH cell (the chicken liver cell line, from ATCC) was cultured in F12/DMEM (Gibco, NY, USA) with 10% FBS (Lonsera, Shanghai, China). The plasmids pcDNA3.1-F2 and pcDNA3.1-F1 kept in our laboratory could efficiently express the fiber-2 and fiber-1 protein of FAdV-4 respectively.

### Antibodies and protein

Chicken sera specific to different serotypes of FAdV were provided by Sinopharm Yangzhou VAC Biological Engineering Co., Ltd. The secondary antibodies including FITC-labelled goat anti-mouse IgG and HRP-labelled rabbit anti-chicken IgY were from Sigma (CA, USA). The purified fusion protein GST-fiber1-S (about 40kD) with the shaft domain of the fiber-1 protein of FAdV-4 (corresponding to the amino acids 79–208 of the fiber-1 protein) was generated in our laboratory.

### Generation of mAbs

The 6-week-old BALB/C mice were immunized with the purified GST-fiber1-S fusion protein four times every 10 days as previously described [16]. At day 3 post the last immunization, the spleen cells from one immunized mouse were collected and fused with SP2/0 cells as previously described [16]. The hybridoma cells secreting antibodies specific to the fiber-1 of FAdV-4 were screened using IFA. In the IFA analysis, the LMH cells infected with FAdV-4 were used as antigen. After the subcloning of the positive hybridoma cells, the mAbs secreted by these positive clones were further identified by IFA, western blot and immunoprecipitation. The ascites of these identified mAbs were prepared and purified as previously described [16]. All the mice studies were performed under ABSL2 facility. At the end of the experiment, all the mice were first anesthetized with isofluorane and then euthanized by cervical dislocation.

### Indirect immunofluorescence assay (IFA)

LMH cells transfected with pcDNA3.1-F1 and pcDNA3.1-F2 respectively or infected with FAdV-4 for three days were fixed with the fixed solution (acetone and ethanol 3:2) for 5 min, and then the IFA was performed according to the previous report [16].

### Immunoprecipitation and immunoblotting

LMH cells transfected with pcDNA3.1-F1 or infected with FAdV-4 for 3 days were lysed in lysis buffer (CST, MA, USA) with protease and phosphatase inhibitors cocktail (CST), and PMSF (Beyotime, Shanghai China). The lysates were collected after centrifugation for 20 min at 12000 rpm and the supernatants were used for western blot and immunoprecipitation as previously described [16].

### Sandwich ELISA

The ELISA plates were coated with the capture mAb (100 μL/well) diluted in 0.1 M carbonate/bicarbonate buffer (pH 9.6) overnight at 4 °C. And the coated ELISA plates were then blocked with PBST (pH 7.4, containing 0.05% Tween-20) with 1% FBS and 5% skimmed milk for 2 h at 37 °C. After the ELISA plates were washed once with PBST, the diluted samples (100 μL/well) in PBST were subsequently added and incubated for 1 h at 37 °C. After the ELISA plates were washed with PBST for four times and the mAb conjugated with HRP (100 μL/well) diluted with 1% skimmed milk in PBST was added to each well. After incubation for 1 h at 37 °C and washing another four times with PBST, the TMB substrate solution (100 μL/well) was added into each well, and the ELISA plates were incubated for 15 min at 37 °C in the dark. 2 M H_2_SO_4_ (50 μL/well) was then added to stop the reaction and the value of OD_450_ was determined by an ELISA plate reader. The condition for the ELISA was optimized by analysis of the OD_450_ value and the positive/negative ratio (P/N) of the samples at different conditions. The cut-off value of the ELISA was evaluated by using 60 FAdV-4 free samples including tissue samples and chicken cloacal swab samples, and was determined by calculating the two folds of the arithmetic mean of these FAdV-4 free samples. For detection of the clinical tissue samples, the liver samples from a diseased chicken flock were homogenated (1 g tissue in 1 mL PBS), and the supernatants from the homogenates were diluted in 1:10 with PBS for the ELISA test.

## Data Availability

The datasets used and/or analysed during the current study are available from the corresponding author on reasonable request.

## Abbreviations

AIV: Avian influenza virus
ALV: Avian leukosis virus
CAV: Chicken infectious anemia virus
FAdV: Fowl adenovirus
IBV: Infectious bronchitis virus
IFA: Indirect immunofluorescent assay
mAb: Monoclonal antibody
MDV: Marek’s disease virus
REV: Avian reticuloendotheliosis virus
